# Enhancement of *Brassica napus* Tolerance to High Saline Conditions by Seed Priming

**DOI:** 10.3390/plants10020403

**Published:** 2021-02-20

**Authors:** Panaiotis M. Stassinos, Massimiliano Rossi, Ilaria Borromeo, Concetta Capo, Simone Beninati, Cinzia Forni

**Affiliations:** 1 Department of Biology, University of Rome Tor Vergata, Via della Ricerca Scientifica, 00133 Rome, Italy; panaiotismario@gmail.com (P.M.S.); capo@uniroma2.it (C.C.); beninati@bio.uniroma2.it (S.B.); 2 PhD Program in Evolutionary Biology and Ecology, Department of Biology, University of Rome Tor Vergata, Via della Ricerca Scientifica, 00133 Rome, Italy; massimiliano87rossi@hotmail.com; 3 Department of Physics, University of Rome Tor Vergata, Via della Ricerca Scientifica, 00133 Rome, Italy; ilaria18scv@hotmail.it

**Keywords:** seed priming, spermidine, rapeseed, salt stress, antioxidant activities, proline

## Abstract

Plants grown in saline soils undergo osmotic and oxidative stresses, affecting growth and photosynthesis and, consequently, the yield. Therefore, the increase in soil salinity is a major threat to crop productivity worldwide. Plant’s tolerance can be ameliorated by applying simple methods that induce them to adopt morphological and physiological adjustments to counteract stress. In this work, we evaluated the effects of seed priming on salt stress response in three cultivars of rapeseed (*Brassica napus* L.) that had different tolerance levels. Seed chemical priming was performed with 2.5 mM spermine (SPM), 5 mM spermidine (SPD), 40 mM NaCl and 2.5 mM Ca (NO_3_)_2_. Primed and not primed seeds were sown on saline and not saline (controls) media, and morphological and physiological parameters were determined. Since SPD treatment was effective in reducing salinity negative effects on growth, membrane integrity and photosynthetic pigments, we selected this priming to further investigate plant salt stress response. The positive effects of this seed treatment on growth and physiological responses were evident when primed plants were compared to not primed ones, grown under the same saline conditions. SPD priming ameliorated the tolerance towards saline stress, in a genotype-independent manner, by increasing photosynthetic pigments, proline amounts and antioxidant responses in all cultivars exposed to salt. These results may open new perspectives for crop productivity in the struggle against soil salinization.

## 1. Introduction

The detrimental and growth-limiting effects of abiotic stress are seriously threatening the sustainability of agriculture [1]. Agricultural lands subjected to salinization are increasing worldwide, and the situation is further aggravated by climate change. Therefore, the understanding of how plants respond to salt and co-occurring stresses can play a major role in stabilizing crop performance under such conditions, as well as in the protection of both natural vegetation and crops [2,3].

*Brassica napus* L. is an extremely important crop being a good producer of oils that can be utilized for both edible oils and biodiesel production [4]. However, this species can be classified as glycophyte, thus with an increasing in soil salinity, growth and yield of this crop can start to decline.

Several tools, such as acclimation [5] and seed priming [6], and the use of plant growth-promoting bacteria (PGPB) [3,7,8] can be utilized to improve plant salt stress tolerance and represent promising strategies. Seed priming is a pre-sowing treatment, consisting of the soaking of the seeds in a priming agent, acting as an elicitor, for a specific period, followed by drying the seeds to avoid radicle emergence. A chemical priming agent can induce abiotic stress to seed, stimulating stress responses and possibly inducing a cross-tolerance to different abiotic stress [9,10]; in other words, the pre-treatment of seeds with chemical agents, by initiating a mild stress-like cue, allows the creation of a “priming memory”, similarly to an acclimation response; consequently, this memory ameliorates the tolerance when the plant is subsequently exposed to different abiotic stress. Priming agents, such as hormones or polyamines (PAs), have been reported to improve the seedling performance in wheat [11] and rice [12]. Such memory acts at phenotypic level [13] and also comprehends epigenetic modifications, changes in gene expression and metabolism, but not changes of DNA sequence.

The profiling of transcriptome shows an increased expression of antioxidant genes, ABA biosynthesis gene, ABA-inducible transcription factors and LEA proteins gene in roots and shoots. Other priming agents (i.e., H_2_O_2_, NO and H_2_S) showed good results in enhancing abiotic stress tolerance. These compounds increased the regulation of genes responsible for redox cell homeostasis and the synthesis of osmoprotectants [14]. PEG 6000, used as priming agent in rapeseed, enhanced the expression of the pyrroline-5-carboxylate synthetase (P5CSA) gene (involved in proline synthesis), the regulation of MYB and the production of transcription factors such as ERF/AP2 and NAC domain-containing proteins [15].

Since the induction of stress tolerance depends on both species and priming agent, a successful application of seed priming needs a screening to detect the best combination of priming agent and plant species. The aim of this work was to seek the possibility to improve salinity tolerance in rapeseed cultivars exposed to high salinity, by evaluating the effectiveness of chemical seed priming as a tool to ameliorate plant survival and fitness. Based on previous results [5] and preliminary tests, three rapeseed cultivars with different levels of tolerance/sensitivity to salt have been selected, i.e., SY Saveo (sensitive), Edimax CI (intermediate tolerant) and Dynastie (tolerant). Four different priming agents were applied to the seeds 2.5 mM spermine (SPM), 5 mM spermidine (SPD), 40 mM NaCl and 2.5 mM Ca (NO_3_)_2_ in order to determine the most effective ones. Primed and not primed seeds were sown in media with different levels of salinity, and seedling growth, representing one of the most delicate phases of plant development, was observed. SPD priming treatment was the most effective; therefore, it was selected to further investigate the responses to salt stress in primed seedlings.

## 2. Results

### 2.1. Priming Effects on Seed Germination and Plant Growth in Saline Conditions

*B. napus* is a species that can be quite sensitive to salinity, particularly during the early phases of development. To improve the performance of the plants in the presence of high levels of salinity, we applied different seed priming agents, i.e., 2.5 mM SPM, 5 mM SPD, 40 mM NaCl and 2.5 mM Ca (NO_3_)_2_. Seed priming improved the germination rates in both exposed or not to moderate and high salinity (Table 1).

The growth of primed seedlings was compared to the not primed ones (controls) exposed or not to salt. Saline conditions reduced the growth of both shoots and roots in a manner related to the increasing salinity of the medium (Table 2 and Table 3). A significant reduction in salt negative effect on growth was detected in plants primed with SPD, especially in Edimax and Dynastie cultivars (Table 2). The positive effect of priming was even more evident in roots (Table 3). The most effective agents were SPD and NaCl, which provided the best improvement in root length (SPD in particular) in both low and high saline conditions.

### 2.2. Membrane Injury Index and Photosynthetic Pigments

Salt stress injury can damage the membranes, decrease chlorophylls content and, consequently, photosynthetic activity. Therefore, membrane injury index (MII) and chlorophyll detection can provide useful information about the level of damage caused to the plants. Overall, SPD reduced membrane damage at all salinities (Table 4); a lowering of MII was also observed with SPM and NaCl priming. On the contrary, MII levels increased in plants primed with Ca(NO_3_)_2_.

Generally, SPD priming increased the chlorophyll amount in the plants, exposed or not to salt (Table 5). While, in calcium nitrate primed samples, the chlorophylls were significantly lower than the controls of Sy Saveo and Dynastie grown at 40 and 160 mM salt concentrations (Table 5). Carotenoid amount was higher in plants primed with SPD, in particular at 160 mM NaCl (Table 6). Vice versa, Ca(NO_3_)_2_ priming decreased the amount of carotenoids in all cultivars at high salt concentration (Table 6).

### 2.3. Effects of SPD Priming on Antioxidant and Osmotic Responses

**Figure 1:**
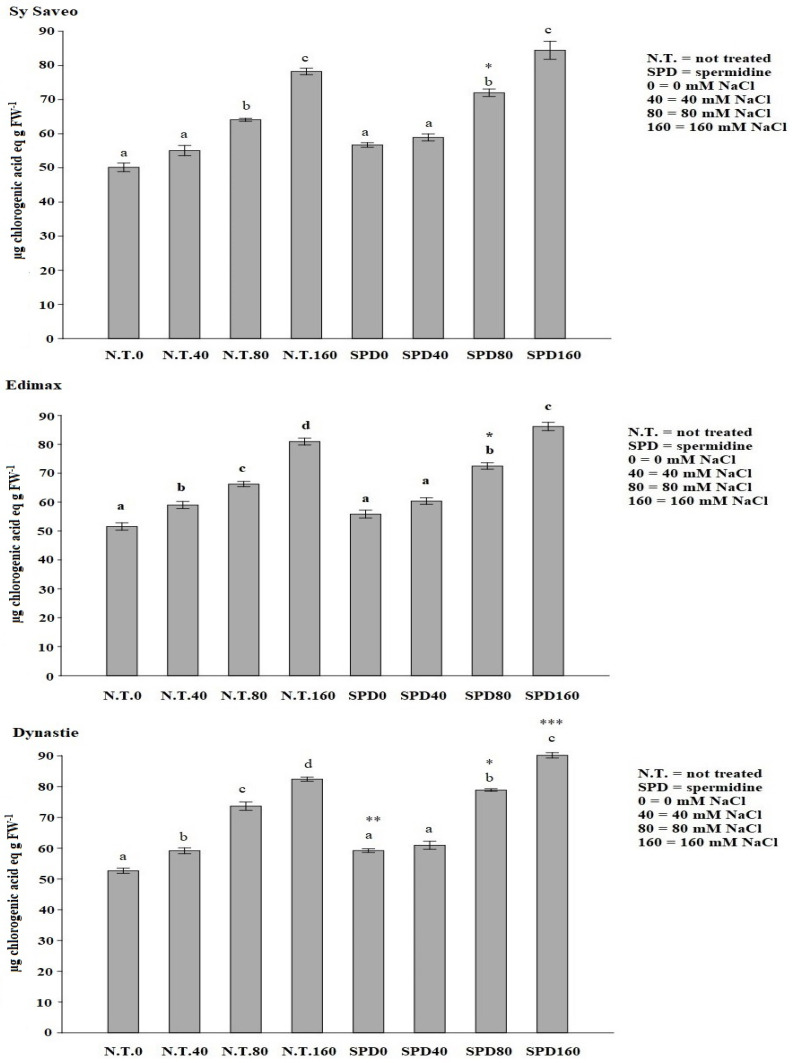
Effect of spermidine priming on total phenol content of rapeseed cultivars exposed to saline conditions. Data are expressed as means ± SE (*n* = 3). Mean values in the column marked by different letters are significantly different within the same group (*p* ≤ 0.05; ANOVA and Tukey–Kramer test). Significant differences between groups are reported as * *p* < 0.05; ** *p* < 0.01; *** *p* < 0.001.

**Figure 2:**
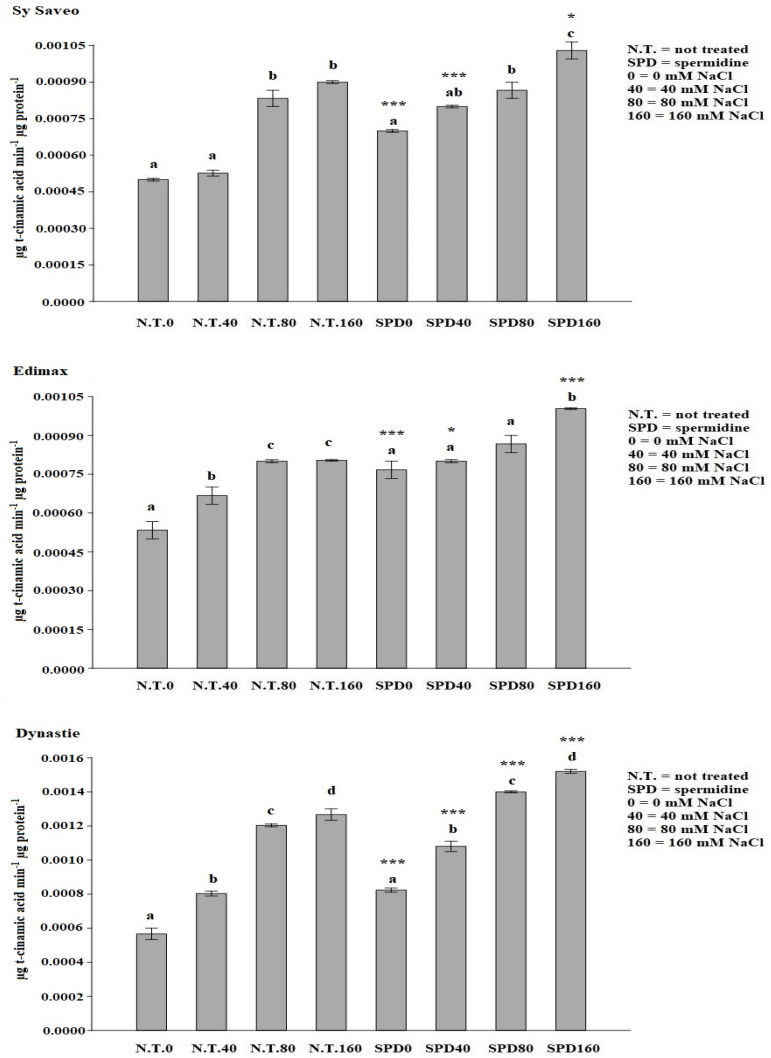
Effect of spermidine priming on PAL activity of rapeseed cultivars exposed to saline conditions. Data are expressed as means ± SE (*n* = 3). Mean values in the column marked by different letters are significantly different within the same group (*p* ≤ 0.05; ANOVA and Tukey–Kramer test). Significant differences between groups are reported as * *p* < 0.05; *** *p* < 0.001.

The priming with SPD was the most effective in all cultivars, showing genotype independence; therefore, we decided to further investigate the effect of this PA in counteracting the toxicity of salt, especially in eliciting antioxidant and osmotic responses of the plants. The latter may represent the key to understanding some of the effects of SPD priming. The synthesis of phenolics and antioxidant enzyme activities counteract the overproduction of ROS, consequent to salt exposure. An increase in the quantity of phenolic compounds was detected in the primed cultivars with respect to the not primed controls with significant differences detected at the different salinity levels (Figure 1). The increase of the synthesis of phenolic compounds was related to PAL activity (Figure 2) that showed an enhancement in primed plants, particularly in Dynastie (Figure 2).

**Figure 3:**
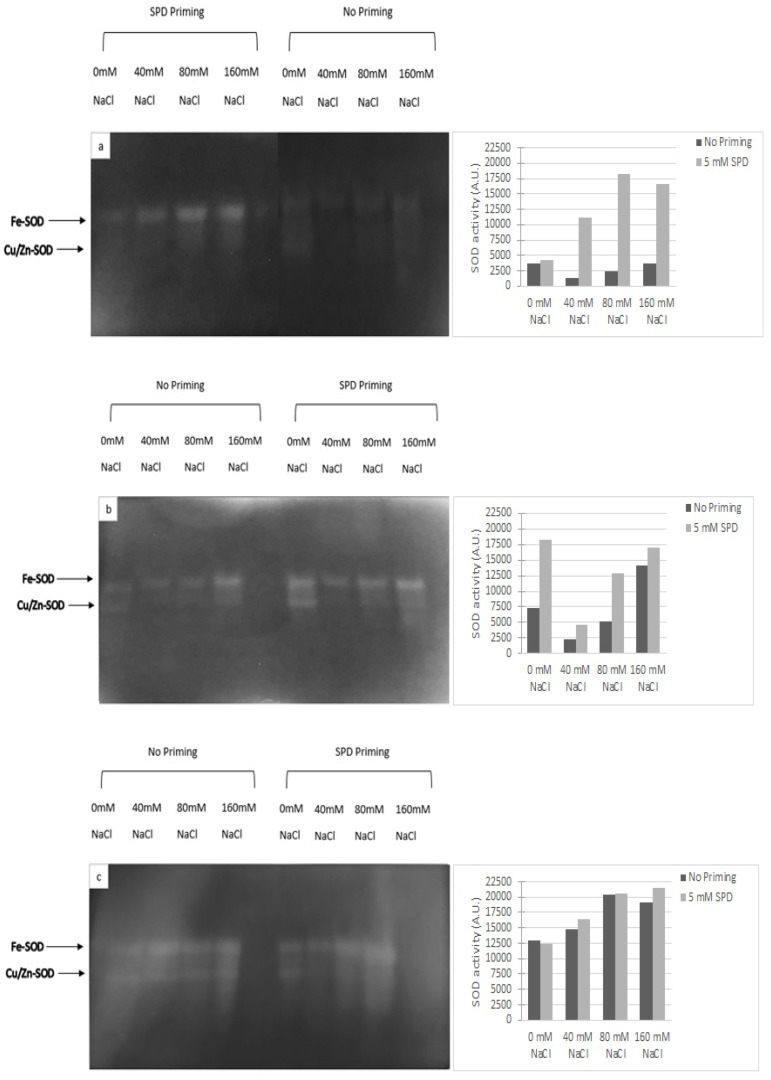
Effect of spermidine priming on SOD activity of rapeseed cultivars exposed to saline conditions ((**a**): Sy Saveo, (**b**): Edimax, (**c**): Dynastie).

The enzymatic antioxidant response was evaluated by determining SOD activity. The latter increased in primed plants with respect to the not primed ones at all NaCl concentrations (Figure 3). Dynastie samples showed small differences in activity between primed and not primed plants (Figure 3).

**Figure 4:**
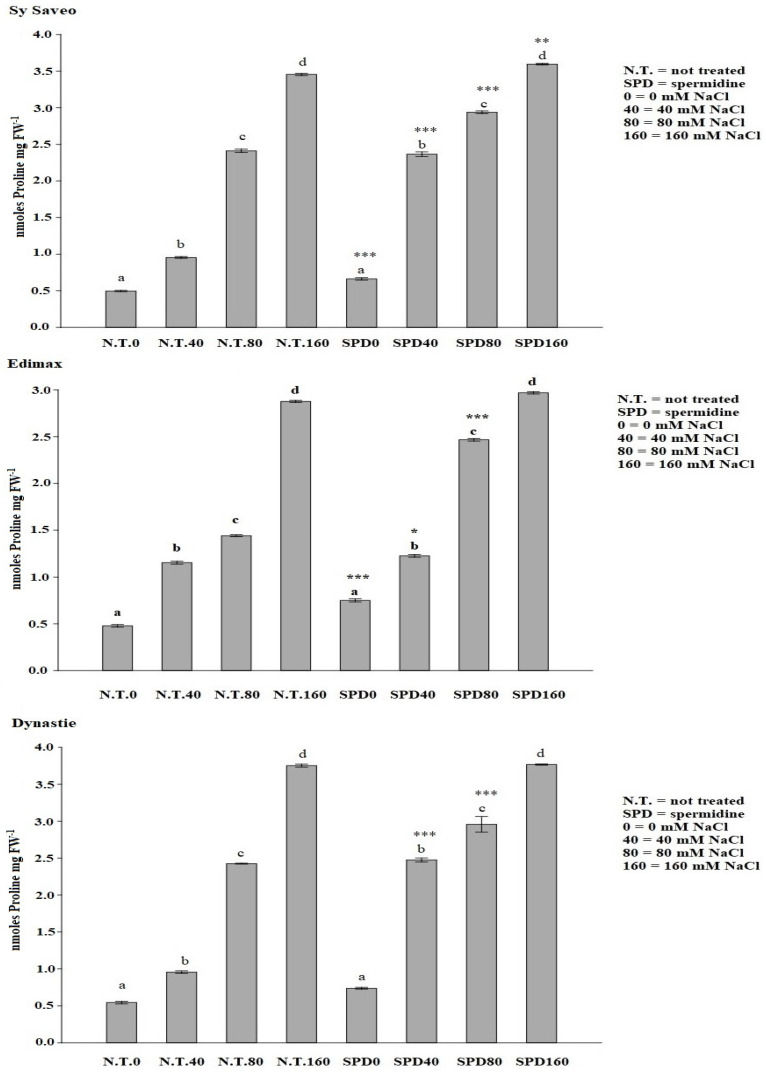
Effect of spermidine priming on proline amount of rapeseed cultivars exposed to saline conditions. Data are expressed as means ± SE (*n* = 3). Mean values in the column marked by different letters are significantly different within the same group (*p* ≤ 0.05; ANOVA and Tukey–Kramer test). Significant differences between groups are reported as * *p* < 0.05; ** *p* < 0.01; *** *p* < 0.001.

Moreover, SPD priming induced the synthesis of the osmolyte proline; its amount enhanced significantly in all samples exposed to salt (Figure 4).

## 3. Discussion

In the literature, NaCl is the most common priming agent [16,17,18], able to increase seed germination rate in salt-stressed plants. PAs have been also tested; in plant, PA activity is involved in several growth stages and development, as well as in the responses to biotic and abiotic stresses [5,19]. An essential role has been attributed to SPD for the survival of *Arabidopsis* embryos, whilst SPM plays a role in stress responses [20]. Both SPD and SPM are able to increase transcription of the Betaine aldehyde dehydrogenase 1 (BADH 1) gene, leading to an increased tolerance to salt stress [12].

In our study, we investigated the effects of four different chemical priming solutions on the response to salt of *B. napus*. For this purpose, we selected cultivars with different levels of tolerance, i.e., SY Saveo (sensitive), Edimax CI (moderately tolerant) and Dynastie (tolerant). Meanwhile, different salinity levels of the medium, ranging from non-saline (control) up to very strongly saline [21], were tested, to detect the threshold of tolerance to salt. According to FAO, the salinities used in this work (up to 160 mM NaCl) are high, and glycophytes cannot grow well or even do not grow at all under these conditions.

The tested priming agents increased the germination rates of all rapeseed cultivars with respect to the not primed group at the same salinity. NaCl primed seedlings grew significantly better than not primed at the same salinity, confirming the data of the literature [22,23], but this increase was genotype dependent. Calcium nitrate had a negative effect on plant growth, suggesting an increase in stress, contrarily to the data of Gouveia et al. [24]. Higher growth rates were observed in PA-treated seedlings exposed to stress, confirming literature reports on other species [11,25].

Membranes integrity was affected by the presence of such elevated salinity; nevertheless priming treatments alleviated the symptoms, especially when SPD, SPM and NaCl were used as chemical agents. These results, in agreement with the literature, suggest a role for PAs in stabilizing cell membranes during salt stress [26].

Controversy exists in the literature regarding the effect of salinity on chlorophyll amount. Generally, a decrease in chlorophyll content caused by salinity has been reported by many researchers [22,27,28]. Otherwise, some authors [29,30] affirm that salinity reduces the chlorophyll content in salt-sensitive plants and increases it in salt-tolerant ones. Our data, in the control group, agree with those by Alamgir and Ali [31], which showed an increase in chlorophyll content in rice plants exposed to salt stress. Both PAs increased total chlorophyll content; in particular, SPD priming showed a significant enhancement in all cultivars. Paul and Roychoudhury [12] reported that seed priming with PAs on rice increased the expression levels of RbcS, a gene encoding for RuBisCO. The authors observed that this increase was absent in the not primed plants. This may be a mechanism playing a role also in rapeseed primed with PA.

Since SPD was the most effective priming, showing genotype-independent responses, we decided to further investigate the effects of SPD priming to verify if, after the stimulus, there was a modification in antioxidant responses (i.e., phenolics, SOD activity), PAL activity and osmolyte synthesis (i.e., proline).

To counteract and mitigate the damage caused by the overproduction of ROS, induced by salt stress [32], plants need to increase the presence of active oxygen scavengers (e.g., phenolics, PAs, glutathione, etc.) and the activity of enzymatic protectors (i.e., superoxide dismutase (SOD), catalase (CAT), ascorbate peroxidase (APX) [3,33,34]. In rapeseed, the SPD priming positively influenced the enzymatic and non-enzymatic antioxidant responses. SODs have been described as the first line of defense against ROS, while CAT subsequently detoxifies H_2_O_2_ [35]. Rapeseed seedlings had higher SOD activity because of the salinity exposure; the enzymatic response was even higher in SPD primed plants. Our data confirmed the results obtained by Paul and Roychoudhury [12].

*B. napus* is a species-rich in phenolic compounds [36], which biosynthesis depends on both genetic and environmental factors, i.e., exposure to stressful conditions, and it is related to the activation of the biosynthetic pathway, which key enzyme is PAL. Our data showed that SPD priming enhanced the activity of PAL, as well as phenolics production, in agreement to the reports on salt-stressed and PA-primed rice plants [12] and of *Salvia miltiorrhiza* Bunge [37].

The synthesis of osmolytes is one of the strategies widely adopted by plants in response to environmental stress [3,38]. Proline, besides being one of the osmolytes acting in osmotic adjustment, it can also be a reservoir of both energy and nitrogen to be utilized under stress conditions. Increased proline contents were detected not only upon stress conditions, but also after ABA and PA treatments [39]. SPD seed priming can influence significantly osmotic adjustment by enhancing proline content in a manner related to salinity, in agreement with the results obtained by Sheteiwy et al. [40].

## 4. Materials and Methods

All reagents were analytical grade or equivalent and purchased from Merck or Sigma-Aldrich, unless otherwise stated. In each set of experiments, all working solutions were prepared immediately before use from stock solutions.

### 4.1. Plant Growth Conditions

The seeds of rapeseed cultivars used in this project were kindly supplied by Dr. Montanari of the CREA-CI (Centro di Ricerca per le Colture Industriali) of Bologna (Italy). The chosen cultivars were “00” variety, yielding high amounts of oil while retaining low quantities of erucic acid and glucosinolate. Seeds were stored at 4 °C until use.

Seed germination tests in saline conditions were undertaken according to Santangeli et al. [5]. The following cultivars were selected based on salt tolerance: SY Saveo (sensitive), Edimax CI (intermediate tolerant) and Dynastie (tolerant).

Priming is a treatment that hydrates the seed in a specific solution; we primed the seeds with the following compounds: spermine (SPM), spermidine (SPD), NaCl and Ca(NO_3_)_2_. The best concentrations of priming solutions and time of treatment (Table 7) were selected based on preliminary experiments, during which the seeds were observed in order to prevent radicle emergence. At the end of the treatments, they were rinsed with distilled water and then the hydration treatment was stopped, and the seeds were allowed to air dry at room temperature to reach the original moisture content (24 h). Primed and not primed seeds were stored and kept at room temperature until the experiments.

After priming treatments, the seeds were surface sterilized (70% ethanol for 5 min, and then soaked in a solution of 1% NaClO for 1 min) and sown on sterile 1/10 strength Hoagland medium [41] added with 0.5% agar (Santa Cruz Biotechnology). Thirty-five seeds of each cultivar (3 replicates per treatment) were inoculated in glass jars containing 150 mL of medium (11 cm diameter; total capacity 0.5 L) and incubated in the dark for 48 h at 24 °C; they were kept in randomized design until germination was obtained. For salt treatment, NaCl solutions were added to the medium, obtaining different levels of salinity, measured by electrical conductivity (EC): 0 (controls; not saline EC = 1.92 dS/m), 40 mM (moderately saline, EC = 5.04 dS/m), 80 mM (strongly saline, EC = 9.16 dS/m) and 160 mM (very strongly saline, EC = 16.89). Experimental groups were divided as follows: (1) not primed seeds (control); (2) not primed seeds exposed to NaCl; (3) primed seeds; (4) primed seeds exposed to NaCl. Germination rates were recorded.

**Figure 5:**
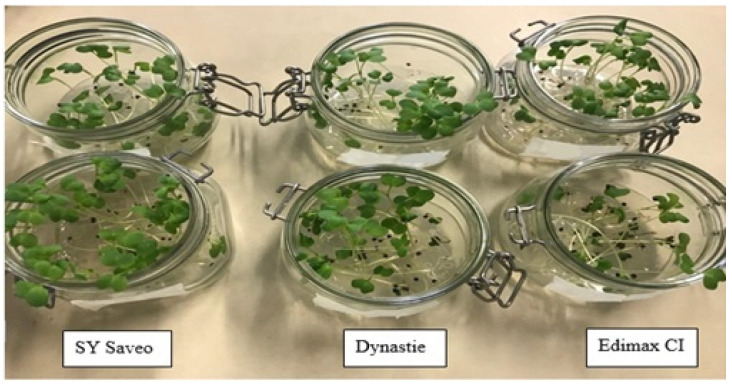
Rapeseed seedlings of the cultivars (Controls below; exposed to 80 mM NaCl above).

The plants were grown for 7 days at 23 ± 2 °C at constant temperature and 48 ± 2% of relative humidity, moved randomly every day, with a photoperiod of 16/8 h, PAR 30 μmoles photons m^−2^ s^−1^ (lamp: 2 × OSRAM, FLUORA t8 36.00 W and 2 × OSRAM, LUMILUX Cool Daylight t 8 36.00 W) (Figure 5).

Seedling growth was evaluated by measuring shoot length and longest root length. Biomass production was determined in plants immediately weighed after harvesting and used for dry weights measurement [5]. Samples (200 mg FW) were frozen by dipping in liquid nitrogen and stored at −80 °C until further analyses.

### 4.2. Membrane Injury Index (MII)

Membrane injury index (MII) was calculated on fresh plant samples by measuring the electrical conductivity (EC) according to Santangeli et al. [5]. Soon after sampling, the seedlings were dipped in milli-Q water in a volume equal to 0.1 mL H_2_O mg FW^−1^. The EC was measured at room temperature with the electrical conductivity meter (Hanna instrument 8333) as follows: after 30 min sample incubation at 40 °C (EC 40°) and then after 10 min sample incubation at 100 °C (EC 100°) in a water bath (Gesellschaft für Labortechnik, GFL). MII was calculated following this formula: MII: EC40°/EC100°*100.

### 4.3. Photosynthetic Pigments

Frozen leaf samples were homogenized with liquid nitrogen using a ceramic mortar and pestle and then dipped in 3 mL of 95% ethanol. The homogenates were incubated in the dark, to avoid chlorophyll degradation, at 4 °C for 1 h, and then centrifugated at 800× *g* for 10 min to remove cell debris. Supernatants were collected and analyzed. To quantify chlorophylls and carotenoids content, the absorbances of the supernatants were measured by a spectrophotometer (Cary 50 Bio. UV-Visible Spectrophotometer) at 664 nm (chlorophyll a), 648.6 nm (chlorophyll b) and at 470 nm (carotenoids).

The concentration of photosynthetic pigments was determined according to Lichtenthaler [42]. The amounts of pigments were expressed as μg mg FW^−1^.

### 4.4. Determination of the Effects of SPD Priming

Basing on the results obtained from the screening with the different priming solutions, we selected the most effective, i.e., SPD priming, for further investigation. Analyses were performed in seedlings that were 7 days old.

#### 4.4.1. Phenolic Compounds

Frozen seedling samples were homogenized in liquid nitrogen, and phenolic compounds were extracted according to Legrand [43]. Homogenization was conducted in ceramic mortars and pestles at 4 °C, in 3 mL of 0.1 N HCl (Sharlau). The samples were incubated for 3 hrs at 4 °C and then centrifuged for 15 min at 8000× *g*. Supernatants were collected, and the pellets were resuspended in 2 mL of 0.1 N HCl and centrifuged again for 15 min at 8000× *g*. Both supernatants were pooled and brought to a final volume of 6 mL with an additional 0.1 N HCl. The total phenolic content was determined by Booker and Miller [44] protocol using Folin–Ciocalteau reagent. Sample absorbance was measured at 724 nm (VARIAN Cary 50 Bio). The concentration of phenolic compounds was calculated according to a calibration curve of chlorogenic acid (CA) (Alfa Aesar) carried out with solutions of 10 μgmL^−1^, 20 μgmL^−1^, 50 μgmL^−1^, 100 μgmL^−1^ (y = 0.0013x − 0.0109; R^2^ = 0.99). The total phenolic content was calculated as μg chlorogenic acid equivalent g FW^−1^.

#### 4.4.2. Proline Content

Proline and total amino acids were extracted using the procedure reported by Santangeli et al. [5]. Seedling samples (50 mg FW) were heated at 55 °C for 20 min in 95% ethanol. After heating, 500 μL of extract was added to 1 mL of 1% (*w*/*v*) ninhydrin (2,2-dihydroxyindane-1,3-dione) dissolved in a mixture of 60% (*v*/*v*) acetic acid (Fluka) and 20% (*v*/*v*) ethanol (Fluka); the reaction mixture was protected from the light and heated again at 95 °C for 20 min. The samples were centrifuged for 1 min at 7000× *g*, and the supernatant was collected for proline detection. The absorbance was measured at 520 nm using the spectrophotometer (VARIAN Cary 50 Bio). Proline concentration was determined from a standard curve made with solutions of L-proline ranging from 0.1 to 1 mM (y = 0.0104x + 0.0294; R^2^ = 0.99). Data are expressed as nmoles proline mg FW^−1^.

#### 4.4.3. Determination of Enzymatic Activities

The enzymatic activities were determined in extracts from frozen samples (200 mg FW) homogenized with ceramics mortars and pestles. The protein content of the extracts was determined by Bradford method [45]. A calibration curve with bovine serum albumin (BSA) (1.25, 2.5, 5 and 10 μg mL^−1^) (y = 0.0468x − 0.021; R^2^ = 0.998) was used to determine the total protein concentration. Phenylalanine ammonia-lyase (PAL, EC 4.3.1.24) was extracted according to the method described by Corsi et al. [46]. Briefly, samples were homogenized in 1% Polyvinylpolypyrrolidone (PVPP) to precipitate the phenolic compounds. The homogenates were re-suspended in 5 mL acetone 80% and 5 mM β-mercaptoethanol and then centrifuged for 10 min at 3000× *g* at 4 °C. The pellets were totally dried under vacuum (Savant), and then suspended in 3 mL of 50 mM sodium-borate buffer (pH 8.8) and centrifuged at 3000× *g* for 10 min at 4 °C. The reaction mixture (3 mL), composed by 1.2 µM L-phenylalanine in 0.05 M sodium-borate buffer (pH 8.8) and crude extract, was incubated at room temperature for 1 h. Enzymatic reaction was stopped by adding 25% (*w*/*v*) trichloroacetic acid. The absorbance of supernatant was detected at 290 nm. The enzymatic assay was performed using a calibration curve (y = 0.1621x + 0.1426; R^2^ = 0.996) of trans-cinnamic acid as standard. The enzyme activity was expressed as μg t-cinnamic acid min^−1^ μg protein^−1^.

Superoxide dismutase (SOD) (EC 1.15.1.1) activity was determined in extracts from frozen leaves (200 mg FW) by NPAGE (native polyacrylamide gel electrophoresis) according to Santangeli et al. [5]. Samples (40 µg proteins) were loaded on native polyacrylamide gel electrophoresis. SOD activity was detected according to Beauchamp and Fridovich [47] and was expressed as Arbitrary Units (A.U.) that correspond to the pixel density of each lane obtained by the program ImageJ.

### 4.5. Statistical Analysis

Data are expressed as mean ± standard error (SE). One-way analysis of variance (ANOVA) was performed with Past 7.0. The Tukey–Kramer method was used to assess the difference of significance among groups. All analyses were considered significant at *p* ≤ 0.05 within each treatment group. When comparing inoculated groups with non-inoculated ones, the significance was *** *p* < 0.001; ** *p* < 0.01; * *p* < 0.05.

## 5. Conclusions

In rapeseed, seed priming can be a tool to increase salt tolerance, ensuring significant enhancement of plant fitness. The positive effect of SPD priming was genotype independent and was observed even at high soil salinity. Different biochemical strategies are adopted by rapeseed primed with SPD to mitigate the negative effects of salt stress. Overall, the data provide compelling evidence to confirm that priming can be very effective to promote crop establishment and survival in saline conditions, thus deserving further study.

## Figures and Tables

**Table 1:** Effect of different priming agents on seed germination (%) of rapeseed cultivars exposed to different saline conditions: 0 mM NaCl (C), 40 mM NaCl (40), 80 mM NaCl (80), 160 mM NaCl (160). Data are expressed as means ± SE (*n* = 3). Mean values in the column marked by different letters are significantly different within the same group (*p* ≤ 0.05; ANOVA and Tukey–Kramer test). Significant differences between groups are reported as * *p* < 0.05; ** *p* < 0.01; *** *p* < 0.001.

Germination (%)					
Cultivars	40 mM NaCl	5 mM SPD	2.5 mM SPM	2.5 mM Ca(NO_3_)_2_	No Priming
Sy Saveo C	98 ± 1 a	100 a *	98 ± 0.88 a	98 ± 0.88 a	95 ± 0.33 a
Sy Saveo 40	96 ± 0.62 ab ***	99 ± 1.13 a ***	97 ± 0.13 a ***	97 ± 0.1 a ***	8 ± 0.95 b
Sy Saveo 80	94 ± 0.6 ab ***	98 ± 0.88 a ***	96 ± 0.7 a ***	87 ± 1.15 b	84 ± 0.66 b
Sy Saveo 160	93 ± 0.33 b ***	92 ± 1 b ***	91 ± 0.88 b ***	85 ± 1.15 b **	77 ± 1.45 c
Edimax C	98 ± 1.1 a **	93 ± 0.57 a	93 ± 0.63 a	98 ± 1.58 a **	91 ± 1 a
Edimax 40	96 ± 0.65 a **	88 ± 1 a	89 ± 1 a	94 ± 0.56 ab *	88 ± 1 a
Edimax 80	91 ± 1 b ***	88 ± 1 a **	84 ± 0.5 b	93 ± 0.16 ab ***	82 ± 1.1 b
Edimax 160	90 ± 0.24 b ***	79 ± 1.13 b	75 ± 1.07 c *	91 ± 1.1 b ***	80 ± 1.82 b
Dynastie C	100 a **	100 a **	100 a **	100 a **	96 ± 0.53 a
Dynastie 40	99 ± 1 ab	98 ± 1 a	95 ± 1.06 b	99 ± 1 a	95 ± 1.23 a
Dynastie 80	97 ± 0.33 b **	97 ± 0.19 a **	94 ± 0.63 b	96 ± 0.52 b **	93 ± 0.47 a
Dynastie 160	93 ± 1.47 c ***	90 ± 1.85 b ***	80 ± 2.13 c	90 ± 1.82 c ***	81 ± 1 b

**Table 2:** Effect of different priming agents on shoot length (cm) of rapeseed cultivars exposed to different saline conditions: 0 mM NaCl (C), 40 mM NaCl (40), 80 mM NaCl (80), 160 mM NaCl (160). Data are expressed as means ± SE (*n* = 8). Mean values in the column marked by different letters are significantly different within the same group (*p* ≤ 0.05; ANOVA and Tukey–Kramer test). Significant differences between groups are reported as * *p* < 0.05; ** *p* < 0.01; *** *p* < 0.001.

Shoot Length (cm)					
Cultivars	40 mM NaCl	5 mM SPD	2.5 mM SPM	2.5 mM Ca(NO_3_)_2_	No Priming
Sy Saveo C	8.4 ± 0.3 a	9.8 ± 0.3 a *	7.5 ± 0.3 a	8.5 ± 0.3 a	8.2 ± 0.4 a
Sy Saveo 40	6.3 ± 0.2 b	5.9 ± 0.2 b	6.1 ± 0.5 a	6.4 ± 0.2 b	5.6 ± 0.2 b
Sy Saveo 80	5.3 ± 0.2 c	4.8 ± 0.2 b	3.8 ± 0.4 b *	5.6 ± 0.2 b	4.9 ± 0.2 b
Sy Saveo 160	1.6 ± 0.1 d	1.9 ± 0.1 c	1.9 ± 0.1 c	1.8 ± 0.2 c	1.4 ± 0.1 d
Edimax C	8.7 ± 0.3 a	9.7 ± 0.3 a **	8 ± 0.3 a	8.2 ± 0.2 a	8.5 ± 0.3 a
Edimax 40	6.5 ± 0.2 b	6.1 ± 0.1 b	6.9 ± 0.4 b *	6.1 ± 0.2 b	5.8 ± 0.2 b
Edimax 80	5.4 ± 0.1 c	5.8 ± 0.3 b **	4.3 ± 0.4 c	5.1 ± 0.3 b	4.7 ± 0.1 c
Edimax 160	2.3 ± 0.3 d *	2.1 ± 0.1 c	2 ± 0.1 d	2.3 ± 0.3 c	1.6 ± 0.2 d
Dynastie C	9.2 ± 0.2 a	10.4 ± 0.3 a **	9 ± 0.4 a	8.4 ± 0.2 a	8.9 ± 0.2 a
Dynastie 40	7.1 ± 0.2 b	7.5 ± 0.3 b **	7.8 ± 0.3 b **	6.6 ± 0.3 b	6.4 ± 0.1 b
Dynastie 80	5.5 ± 0.2 c	6.2 ± 0.3 c **	5 ± 0.2 c	5.2 ± 0.2 c	4.9 ± 0.1 c
Dynastie 160	2.7 ± 0.3 d	3 ± 0.2 d *	2.4 ± 0.2 d	2.6 ± 0.2 d	2.2 ± 0.2 d

**Table 3:** Effect of different priming agents on root length (cm) of rapeseed cultivars exposed to different saline conditions: 0 mM NaCl (C), 40 mM NaCl (40), 80 mM NaCl (80), 160 mM NaCl (160). Data are expressed as means ± SE (*n* = 8). Mean values in the column marked by different letters are significantly different within the same group (*p* ≤ 0.05; ANOVA and Tukey–Kramer test). Significant differences between groups are reported as * *p* < 0.05; ** *p* < 0.01; *** *p* < 0.001.

Root Length (cm)					
Cultivars	40 mM NaCl	5 mM SPD	2.5 mM SPM	2.5 mM Ca(NO_3_)_2_	No Priming
Sy Saveo C	10.3 ± 0.4 a ***	10.6 ± 0.4 a ***	9.3 ± 0.7 a ***	11.5 ± 0.3 a ***	7.3 ± 0.6 a
Sy Saveo 40	8.6 ± 0.3 b ***	8.1 ± 0.4 b ***	7.7 ± 0.7 b ***	7 ± 0.3 b **	5.6 ± 0.6 b
Sy Saveo 80	5.6 ± 0.5 c **	7.1 ± 0.6 b	4.8 ± 0.7 c **	5.5 ± 0.4 c **	7.1 ± 0.4 a
Sy Saveo 160	4.4 ± 0.2 d ***	5.8 ± 0.2 c ***	1.9 ± 0.2 d	3.5 ± 0.2 d **	1.8 ± 0.2 c
Edimax C	10.6 ± 0.3 a **	12.3 ± 0.5 a ***	10.7 ± 0.5 a **	9.6 ± 0.3 a	8.9 ± 0.3 a
Edimax 40	9.0 ± 0.3 b **	8.8 ± 0.3 b **	8.6 ± 0.5 b *	8.5 ± 0.3 b *	7.5 ± 0.2 b
Edimax 80	6.2 ± 0.4 c	6.9 ± 0.4 c *	6.1 ± 0.4 c	5.1 ± 0.3 c *	6.2 ± 0.2 c
Edimax 160	4.9 ± 0.2 d ***	6.3 ± 0.3 c ***	2.6 ± 0.3 d	3.8 ± 0.3 d *	2.2 ± 0.3 d
Dynastie C	10.8 ± 0.3 a *	13.6 ± 0.2 a ***	11.3 ± 0.3 a **	9.2 ± 0.2 a	9.8 ± 0.3 a
Dynastie 40	9.3 ± 0.3 b **	9.7 ± 0.3 b **	9.8 ± 0.3 b **	8.9 ± 0.2 a *	7.9 ± 0.2 b
Dynastie 80	6.5 ± 0.3 c	8.3 ± 0.3 c **	7.4 ± 0.2 c *	5.5 ± 0.3 b *	6.6 ± 0.2 c
Dynastie 160	5.4 ± 0.2 d ***	7.1 ± 0.2 d ***	4.6 ± 0.3 d **	2.5 ± 0.3 c	2.7 ± 0.2 d

**Table 4:** Effect of different priming agents on membrane injury index (%) of rapeseed cultivars exposed to different saline conditions: 0 mM NaCl (C), 40 mM NaCl (40), 80 mM NaCl (80), 160 mM NaCl (160). Data are expressed as means ± SE (*n* = 8). Mean values in the column marked by different letters are significantly different within the same group (*p* ≤ 0.05; ANOVA and Tukey–Kramer test). Significant differences between groups are reported as * *p* < 0.05; ** *p* < 0.01; *** *p* < 0.001.

Membrane Injury Index (MII %)					
Cultivars	40 mM NaCl	5 mM SPD	2.5 mM SPM	2.5 mM Ca(NO_3_)_2_	No Priming
Sy Saveo C	18 ± 0.2 a	15 ± 0.87 a **	19 ± 0.33 a	21 ± 0.3 a *	19 ± 0.47 a
Sy Saveo 40	25 ± 0.24 b **	26 ± 1.53 b ***	33 ± 1.46 b	30 ± 0.96 b *	33 ± 0.29 b
Sy Saveo 80	43 ± 0.88 c *	33 ± 1.11 c ***	43 ± 0.1 c *	51 ± 1.35 c **	46 ± 1.13 c
Sy Saveo 160	46 ± 0.41 c ***	40 ± 0.74 d ***	48 ± 0.26 d ***	61 ± 1.91 d **	55 ± 2.12 d
Edimax C	18 ± 1.31 a	13 ± 1.84 a **	17 ± 0.13 a	24 ± 1.41 a **	18 ± 1.38 a
Edimax 40	24 ± 1.05 b **	23 ± 0.92 b **	31 ± 0.94 b *	32 ± 1.2 b **	28 ± 1.06 b
Edimax 80	44 ± 0.82 c **	31 ± 0.31 c ***	41 ± 0.29 c *	49 ± 1.35 c ***	39 ± 1.09 c
Edimax 160	55 ± 0.21 d	43 ± 2.09 d ***	46 ± 0.11 d ***	63 ± 3.27 d ***	55 ± 2.33 d
Dynastie C	18 ± 1.53 a	13 ± 1.25 a **	20 ± 0.24 a	25 ± 1.18 a *	20 ± 0.91 a
Dynastie 40	25 ± 0.86 b **	20 ± 0.14 b ***	29 ± 0.89 b	33 ± 0.23 b *	29 ± 1.2 b
Dynastie 80	43 ± 0.4 c ***	23 ± 0.38 b ***	40 ± 0.99 c **	55 ± 0.94 c ***	36 ± 0.3 c
Dynastie 160	56 ± 1.01 d **	32 ± 0.9 c ***	47 ± 1.31 d *	61 ± 1.07 d ***	51 ± 1.12 d

**Table 5:** Effect of different priming agents on total chlorophyll content (μg g FW^−1^) of rapeseed cultivars exposed to different saline conditions: 0 mM NaCl (C), 40 mM NaCl (40), 80 mM NaCl (80), 160 mM NaCl (160). Data are expressed as means ± SE (*n* = 8). Mean values in the column marked by different letters are significantly different within the same group (*p* ≤ 0.05; ANOVA and Tukey–Kramer test). Significant differences between groups are reported as * *p* < 0.05; ** *p* < 0.01; *** *p* < 0.001.

Chlorophylls (µg g FW^−1^)					
Cultivars	40 mM NaCl	5 mM SPD	2.5 mM SPM	2.5 mM Ca(NO_3_)_2_	No Priming
Sy Saveo C	91.4 ± 0.87 a	95.54 ± 0.73 a	91.2 ± 1.54 a	89.02 ± 1.18 a	91.33 ± 0.8 a
Sy Saveo 40	93.14 ± 1.17 a	94.74 ± 1.42 a	95.8 ± 0.97 a	90.31 ± 0.42 a *	94.04 ± 0.95 a
Sy Saveo 80	93.82 ± 1.09 a	98.59 ± 1.47 a ***	98.03 ± 2.03 b **	92.46 ± 1.23 a	92.93 ± 1.36 a
Sy Saveo 160	91.63 ± 0.9 a *	102 ± 0.36 b ***	99.11 ± 1.27 bc **	90.38 ± 0.66 a *	94.3 ± 1.16 a
Edimax C	93.82 ± 0.51 a	95.95 ± 0.65 a *	94.24 ± 1.24 a	91.38 ± 0.88 a	93.25 ± 0.96 a
Edimax 40	95.65 ± 1.22 a	101 ± 0.98 bd **	96.89 ± 1.35 a	93.17 ± 0.82 ab	95.76 ± 1.43 a
Edimax 80	93.89 ± 0.54 a	103.9 ± 0.9 bc ***	99.04 ± 1.66 a **	94.77 ± 0.71 b	94.06 ± 0.77 a
Edimax 160	93.23 ± 0.93 a *	104.15 ± 1 cd ***	100 ± 1.22 a *	94.41 ± 0.62 ab	96.38 ± 0.83 a
Dynastie C	97.39 ± 0.45 a	98.47 ± 0.55 a	98.35 ± 0.72 a	95.77 ± 0.46 a	98.46 ± 0.54 a
Dynastie 40	98.28 ± 0.48 a	105.8 ± 0.6 bc **	101.44 ± 0.98 a	95.86 ± 0.57 a *	98.84 ± 1.42 a
Dynastie 80	97.31 ± 0.9 a	107.6 ± 1.4 cd ***	99.87 ± 0.62 a	96.08 ± 1.06 a	97.56 ± 0.87 a
Dynastie 160	97.37 ± 0.72 a *	107.25 ± 0.15 d **	103.17 ± 0.82 a	96.5 ± 0.24 a **	101.15 ± 1 a

**Table 6:** Effect of different priming agents on total carotenoid content (μg g FW^−1^) of rapeseed cultivars exposed to different saline conditions: 0 mM NaCl (C), 40 mM NaCl (40), 80 mM NaCl (80), 160 mM NaCl (160). Data are expressed as means ± SE (*n* = 8). Mean values in the column marked by different letters are significantly different within the same group (*p* ≤ 0.05; ANOVA and Tukey–Kramer test). Significant differences between groups are reported as * *p* < 0.05; ** *p* < 0.01; *** *p* < 0.001.

Carotenoids (µg g FW^−1^)					
Cultivars	40 mM NaCl	5 mM SPD	2.5mM SPM	2.5 mM Ca(NO_3_)_2_	No Priming
Sy Saveo C	38.36 ± 0.11 a	38.1 ± 0.16 a	37.7 ± 0.16 a	37.71 ± 0.07 a	37.93 ± 0.08 a
Sy Saveo 40	38.31 ± 0.12 a	38.54 ± 0.06 a	38.05 ± 0.2 a	38.06 ± 0.17 a	38.17 ± 0.17 a
Sy Saveo 80	37.94 ± 0.13 a	38.9 ± 0.14 b	38.3 ± 0.14 a	37.18 ± 0.23 b *	38.2 ± 0.18 a
Sy Saveo 160	37.85 ± 0.1 a	39.05 ± 0.11 c **	38.73 ± 0.18 b *	36.48 ± 0.18 c *	37.83 ± 0.08 a
Edimax C	38.56 ± 0.12 a	38.85 ± 0.14 a	38.38 ± 0.17 a	38.06 ± 0.17 ab	38.22 ± 0.17 a
Edimax 40	38.54 ± 0.06 a	38.8 ± 0.17 a	38.51 ± 0.1 ab	38.44 ± 0.06 b	38.18 ± 0.27 a
Edimax 80	38.5 ± 0.18 a	39.2 ± 0.16 a *	38.65 ± 0.18 ab	37.77 ± 0.09 ca *	38.55 ± 0.22 a
Edimax 160	38.36 ± 0.09 a	39.29 ± 0.31 a *	39.26 ± 0.21 b *	36.89 ± 0.2 d *	38.16 ± 0.17 a
Dynastie C	39.19 ± 0.11 a	39.37 ± 0.06 ab	38.67 ± 0.15 a	38.66 ± 0.03 a	39.15 ± 0.12 a
Dynastie 40	38.96 ± 0.14 a	39.25 ± 0.11 a	38.68 ± 0.09 a	38.7 ± 0.12 a	39.04 ± 0.07 a
Dynastie 80	38.97 ± 0.16 a	39.52 ± 0.18 ab	39.22 ± 0.07 bc	38.57 ± 0.09 a	39.32 ± 0.19 a
Dynastie 160	37.99 ± 022 b	39.84 ± 0.09 b *	39.46 ± 0.04 c	37.5 ± 0.09 b **	39.08 ± 0.03 a

**Table 7:** Chemical priming treatments of the seeds with the different priming solutions.At the end of the treatments, the seeds were dried at room temperature.

Priming	Treatment (hours)
2.5 mM SPM	4
5 mM SPD	3.5
40 mM NaCl	5
5 mM Ca(NO_3_)_2_	3

## Data Availability

All data presented in this study are available in the article.

## Abbreviations

fresh weight (FW); spermidine (SPD); spermine (SPM); polyamines (PAs); phenylalanine ammonia lyase (PAL); superoxide dismutase (SOD).
